# American Medical Association, Section on Stomatology

**Published:** 1901-10

**Authors:** 


### Reports of Society Meetings.

### AMERICAN MEDICAL ASSOCIATION, SECTION ON STOMATOLOGY.

### DISCUSSION ON PAPER ON "PERIODS OF STRESS AND THEIR DENTAL MARKS," BY DR. JAMES G. KIERNAN, CHICAGO.

(For Dr. Kiernan's paper, see page 680.)

*Dr. Eugene S. Talbot,* Chicago.—Mr. President, I have been interested in this subject for years, and have spent a great many years in the study of these conditions. I never have heard a paper so clearly define those conditions as the one that has been read. Some one has said that the discussion of dental subjects has been exhausted, but a knowledge of the conditions of the mouth, so far as hereditary environment and embryology are concerned, is still embryonic. There are some points which deserve attention. Dentists often remark that if patients were more careful with their teeth they would not decay, and then prescribe a course of treatment handed down for decades. The same old story is told of brushing the teeth, recommending the use of certain preparations, etc. As Dr. Kiernan said, evolution of the teeth is tending towards extinction of many teeth. Decay is the natural process, and all treatment on earth will not save teeth except such needed in the individual. It is not only a personal calamity, but it has become a national calamity. Study of teeth and jaws of the peoples of Europe shows teeth of men deteriorating where the people are not of a nervous tendency, as they are in other countries. Through

Greece, Turkey, Russia, then around through Norway and Sweden, and through France and England, there is evident degeneracy of the jaws. There are no peoples in the world whose teeth are decaying to-day like those of the English people. There are no irregularities of the teeth like those of the English people. In England it has become a national calamity, and the authorities have appointed committees and have furnished money to investigate the cause of decay of the teeth. All attention and the best methods of treatment will not save them.

There is another condition of the jaws and teeth that Dr. Kiernan has discussed,—the evolution of the second set of teeth. Dentists think they are able to treat pyorrhœa alveolaris or gingivitis, and some claim to cure every case. With them it is simply a matter of local application, a peculiar sense of touch with instruments, to remove all visible deposits. The condition, however, is atavistic, and the disease is preventable to a certain extent; still, should the individual live long enough, he would lose his teeth. This is a process of osteomalacia and an atavistic condition. As Dr. Kiernan has said, change of the teeth is going on continually. Where one tooth is shed after it has done its duty, another takes its place. Where the temporary teeth are shed by absorption, the second set takes its place. This absorption goes on and the teeth are lost in time. Not in all cases, but in the majority of cases, if the person lives long enough. Dr. Kiernan showed you a picture of degeneracy and arrest of development. This is a picture where the child has grown to manhood, yet remained arrested in development. If the internal structures of the body were examined, arrest of development is evident. All have seen such cases, since they are very common. This particular condition illustrated has arrest of development of the jaws. Dr. Kiernan remarks that temporary structures are changing from generation to generation. For this reason dentists should be careful in regulating teeth, especially in children of from one to twelve years of age. These children are in the condition shown. In this neurotic condition, this arrest of development, the nervous system is very easily involved, and also other organs of the body. Children are often invalided for years by having their teeth regulated, and dentists should be very careful on that point. Another point about regulating teeth in such patients needs attention. Dentists cannot tell just exactly how it comes about, but where the teeth are moved by springs, as is often done by dentists, and by ligatures and all kinds of appliances, they cannot be held perfectly firm. Later in life occurs an absorption of the alveolar process at this very point, due to osteomalacia or alveolar absorption. Gingivitis sets in from regulation of the teeth, the jaws are never restored to health, and the absorption goes on until the teeth become loose and are lost.

I want to call your attention to the four-and-one-half-months-old embryo. All have seen children that have the appearance of age, with little hairs upon the face and ears, with wrinkles in the face, the hair frequently gray in spots, the skin drawn up, and the fingers long and slender. In such patients dentists should be very careful in the regulation of the teeth and in all operations. They always have irregularities, and the greatest care is necessary because of the unbalanced nervous system.

*Dr. Vida A. Latham,* Rogers Park, Ill.—I want to express a word of thanks to Dr. Kiernan for his very able paper. I think it is a good thing to have the tension made clear on pathological principles. Many teachers have made the statement that it is absurd to suppose that the changes are made through the nerve-functions. They can occur through tension, perhaps not permanently, but it is one of the factors underlying this development, or it may be through a case of retrogression. When we think of the enormous amount of force that goes on in the development of the teeth coming through four dental cycles, and of the congestion which involves the vasomotor and vasoconstrictor systems, we may come to the conclusion that it is duê to the period of first stress. I have heard men advance the argument that pathological stress is absurd, that it cannot come from such a source.

*Dr. G. V. I. Brown,* Milwaukee, Wis.—I cannot say very much on this subject without encroaching on the paper that I expect to read by and by. I shall be obliged to disagree with our secretary, however, concerning the regulation of teeth, because I have to begin to regulate the teeth of these youngsters before they are entirely destroyed. In regard to what he says of the disastrous results following the regulation of teeth in children and young persons, I think it was rather the fault of the method than the age of the patient, and I am convinced that where that continual absorption went on it was due to the effect of occlusion of the jaws on account of malposition, which caused an irritation of the tissue around the mouth.

*Dr. James G. Kiernan,* Chicago.—I only wish to correct a little terminology which I think might be desirable in this Section. There is perhaps no such word as "degeneracy." Now, the question is whether there is degeneracy or whether it is a disappearance of certain parts for the benefit of the organism as a whole. It is true a great many changes do occur in this way for the benefit of the organism as a whole. For example, the human teeth in number are disappearing. There is very good reason to believe that man at one time possessed at least two extra incisors. There is also very good reason for believing that the wisdom-tooth is tending to disappear. There is also good reason to believe that the human tooth is becoming microdont rather than macrodont, rather smaller than larger. Now, this is only along the general line of evolution with reference to the face and jaws. The human jaw itself with all its beauty, the human face with all its beauty, is degenerate. For biting purposes and chewing purposes it is not as valuable as the jowl of the lower races. It is an embryonic feature. This space has been made for the benefit of the brain space. The brain has been absorbing more and more space at the expense of the bones of the face. Therefore, in dealing with this question of irregularities it would be wise to look at it in certain cases as a normal process.

I throw out this subject for your opinion because I am not a dental scientist, but I think in certain cases it would be wise to study the teeth not only from the point of regulation, but also from the stand-point of removal a little more than is done. No human hand can turn back the clock of time and make a high type of man and restore the jaw of his prehistoric ancestor, who lived on food in which there was an enormous waste. The native or prehistoric races had beautiful teeth, but those teeth could not exist with the environment of the civilized races. Civilized man does not waste his food so much; he does not need to do so much chewing and tearing. In the only animal that can be compared with man, the dog, similar changes are taking place, and these changes are coincident with the changes taking place in civilized man. We have another factor that influences these changes. There are many race types with different jaws and different teeth. No races are pure considered from this stand-point; all races are intermixed. It is safe to say that every one of the English-speaking races has some prehistoric element in it; every one of the Teutonic races and every one of the Euro-African races have the same element in them, as have also the Asiatic. Now, these races have different types of jaws, different sizes of teeth, and from them comes the so-called Aryan mixture. These people are subjected to a new environment. It is, therefore, the duty of modern dentists to study how far in one case removal of the teeth in a certain type might be a benefit in correcting cases, and how far in another it might be injurious, and deal with the matter from that standpoint. You cannot deal with it in a general way, but you must take into consideration the individual conditions to which the patient is exposed. We read a great deal about the degeneracy of the teeth. It is usually ascribed to luxury. The degeneracy may be an expression of the advance of the human race. When man gets fewer teeth, then there would not be so much irregularity, and there would not be so much trouble in other directions.

DISCUSSION ON PAPER ON "INFECTIOUS DISEASES," BY DR. ALICE STEEVES, CHICAGO.

(For Dr. Steeves's paper, see page 686.)

*Dr. Vida A. Latham,* Rogers Park, Ill.—This is a subject that is hardly touched upon by dentists, but in connection with the physician we can accomplish a great deal in this direction. Many cities have a quarantine law, and in that way we can control these diseases in large cities; but it is more difficult where a physician is allowed to use his discretion in placarding a house. There is great latitude in that method. If a physician is not perfectly honest and upright, he can probably be persuaded by higher fees or in some other way to favor the family. A man will tell him that his wife goes out in society, that he must go to business, and the result is the physician does not quarantine the father of the family, and he goes to the bank or to his business without hinderance. I have had just such cases, and I have had to scold pretty severely. When a man comes home at night it is natural that the child wants to see its father, and, if possible, he will steal in to it somehow.

I have had two horrible cases of infection of little folks through the non-disinfection of instruments, more especially forceps. In one case that was referred to a specialist a syphilitic lesion was caused in the upper jaw through the use of unclean instruments. I did not care to bear a malpractice suit, and that case was referred to a specialist. In dental schools how many students are taken to see bacteriological cases of syphilis in the oral cavities? They will not see those cases, because they are not pointed out to them. I have seen them, because I am practising medicine and dentistry. I have been in dental schools where they are not shown at all. Unquestionably students ought to study and know these conditions. Most of us if we should see a case would not recognize it. Therefore I hope the teaching of surgical cleanliness will be broader and deeper in all its branches.

*Dr. Geo. T. Carpenter,* Chicago.—I recognize the importance of being able to know these conditions when you come across them. The ordinary practitioner will not recognize these, particularly sypilitic lesions, and it is a very difficult matter, even where he suspects a case, to secure its history. Syphilis is a disease that is covered up, and sometimes the only thing the practitioner can do is to pursue syphilitic treatment in a suspected case, and there will be a response if his surmise is correct.

We cannot be too particular in regard to the absolute importance of sterilizing instruments. Less than a year ago I had a family connection in my office having work done. He spoke of a slight abrasion at the corner of the mouth. I cleansed it and gave it some trivial local treatment, and the incident passed from my notice. At the same time I held the chair of oral surgery in one of our schools. A short time after that a brother, who was a medical student at that time, asked me if I had noticed William's mouth. I told him I had not specially noticed it. He said he wished I would examine it, as he himself was a little alarmed. He had been taking one of the preparations of mercury, and he believed he had secondary syphilis. If a person teaching these things will let them pass from under notice what will the ordinary practitioner do? Consequently I think we should be alive to the existence of these conditions, but, above all, we should pay strict attention to the sterilization of our instruments.

*Dr. A. E. Baldwin,* Chicago.—I am very much interested in anything that pertains to this branch of the work. The statement is made that cleanliness is next to godliness, but I believe that cleanliness is godliness, especially in the dental chair. I do think we sometimes exaggerate the importance of the health departments of our large cities in regard to the transmission of many of these diseases. It is a question as to whether some of these diseases that are called infectious or contagious really are what the name implies. Where these conditions are present in healthy mouths, for instance, tubercle bacilli will be present in the mouths of perfectly healthy people. I suppose the only conclusion arrived at is that we must have a proper condition of the system, so that the resistance is great enough to throw off the effect of this systemic poison. It seems hardly necessary to enlarge upon cleanliness, but I recognize the condition Dr. Carpenter speaks of. It requires a good deal of courage to own up to our shortcomings, but I think if the truth were told by all of us some things fully as bad or even worse might be said than those mentioned, but the only thing to do is to fix the fact in our memory that we must be exceedingly careful in everything that tends to the welfare of our patients, to the absolute cleanliness of our instruments, our hands, and our persons.

*Dr. Jas. G. Kiernan,* Chicago.—The only solution is to point to a rather serious error in Dr. Baldwin's statement. In the first place, two things are necessary, the culture medium for transmitting the particular germ, and the germ. There is probably no healthy individual that has not bacterial germs in his mouth, but, on the other hand, while he is immune under given conditions, those bacteria may be transmitted to another individual and the result be continued indefinitely. The particular medium may be immune, but he continues the danger. That danger, however, I think has been met by the prevailing practice in the case of dentists of using antiseptic washes. There may be certain bacteria in individual mouths, and those bacteria may not gain entrance into the system until after operation. This is the case with the streptococcus, the staphylococcus, and other bacteria, and the fact, as Dr. Baldwin states, of the existence of bacteria in a healthy mouth contains an element of danger greater than is generally recognized.

*Dr. Vida A. Latham.*—I would like to emphasize a point Dr. Kiernan brought out. I think doctors and physicians should pay special attention to the throats of children at school. How many children are allowed out under two weeks with an infectious or contagious disease? In England at least six weeks is required, or until all the scabs have disappeared. Here, of course, it is rather light, but there the mortality is greater. In the case of scarlet fever it may be only a light attack, and the child may be back in school in two weeks. I know a child who had a light attack of scarlet-fever whose mother allowed it to play with other children, putting kid gloves on the hands of the child. Of course, there is danger of infection. I know a friend of mine, a young lady, who went to a house where there was a case of scarlet fever. She was informed that the little girl had been down with scarlet fever. The young lady went home, changed all her clothing, and went to another house; from there she went to a dentist's office, where she had an appointment. That is where contagion comes from. I do not think children should be allowed out as early as they are in Chicago.

*Dr. G. V. I. Brown,* Milwaukee, Wis.—I remember last year a gentleman gave us a very scholarly dissertation upon this subject, and it set me to thinking a good deal. Since then I have tried to do a little missionary work, and during the last year have given at various times before school boards and school committees and any who were interested an illustrated discussion of this subject, in which I tried to enforce the value of care in this direction by having views thrown from the lantern upon the wall illustrating the different germs already described, particularly the diphtheria bacillus, the pneumococcus, and so on. I do not think I can do better at this time than to emphasize again the necessity of doing something more than talking these things over among ourselves. We all know the danger of infection. We are benefited by having the idea that cleanliness is next to godliness impressed upon our minds, but if we would resolve ourselves into a committee of the whole and go out from here and spread the information until we made school boards and those who have charge of the instruction and care of young people recognize the importance of these things, we would be doing a great deal more than we are doing here now. I have tried to make it strong in speaking of diphtheria by calling attention to the fact that Park View had an epidemic of diphtheria, and at least one per cent. of the children had these germs in the mouth. In Milwaukee we have had members of the medical profession during an epidemic examine the mouths and throats of children every morning before school began, but it seems to me that is a good deal like locking the barn door after the horse is stolen, because by the time the lesion is manifest or the disease recognized, the child has already exposed more children than it would be possible for us to compute. The same is true in a large measure of tuberculosis. If one in every seven dies of tuberculosis, as has been stated by Dr. Senn, and others agree with him on that point, and the disease is so prevalent that it defies all our laws of sanitation, it seems to me the practical thing to do is to begin right in the mouth. In by far the majority of cases of affections of the lungs the bacilli are in the mouths continually, and whatever care may be taken of the sputum of such people after it leaves the mouth, a little care taken before would be much better. It is a simple matter for public schools to have a large vessel at the door with some cheap antiseptic and make it obligatory upon pupils to rinse their mouths before entering the school-room. I am certain we would do more to check diphtheria and influenza by attending to these things than we can in any other way. I think it is well worth continually repeating until we are tired of it, or until we finally make the right impression upon the minds of the people in charge of our public institutions. We are not politicians, but if we can arouse the laity without politics by continually reiterating these facts before the people, we shall feel that our labors have not been in vain.

*Dr. A. E. Baldwin,* Chicago.—I just want to say that I do not see that anything Dr. Brown said adds force to what has been stated by Dr. Latham or myself. I think a great deal of harm comes from posting notices. I know they would agree with me in many large cities. Here is a case of a child that has scarlet fever. That child was isolated from the household and no one allowed to see it except the trained nurse; no one else ever went near it. Would it be right to isolate that whole building by putting a placard on the front door? They would not be incautious, they could not be affected at all by being in another part of the house. Does that little card eight by fourteen offer any protection? Not at all; it does nothing of the kind. I agree with Dr. Latham that there should be a rigid enforcement of separation between those affected and those not affected, children as well as adults.

Speaking of what Dr. Brown has said, I do not think tuberculosis is dangerous to us so long as it is in Dr. Brown's mouth. It does not circulate in that way. The only circulation it has is in the air or in food and drink, and the remedy he suggests would do no good in lung difficulties. If you could have any preparation to take into the mouth to destroy tubercle bacilli it would have no effect one-half hour later. Notwithstanding the isolation of these cases, you will find these Klebs-Loeffler bacilli, peculiar to diphtheria, in many mouths that are healthy, and we cannot look into every mouth. I do not want anybody to misunderstand me, I do not want to throw open the door to every diphtheritic case, but there are a great many things that we advocate that are really not as far-reaching as we think they are. I think it is far from proved that many diseases that are called contagious or infectious are really so.

*Dr. G. V. I. Brown.*—I want to say just a word in illustration of one single point in this matter. I think we are generally agreed that we are all talking of what we do not know. In the isolation hospital in Milwaukee during an epidemic of scarlet fever the patients were immediately placed upon a system of treatment which included the disinfection of the mouth with hydrogen dioxide and oxygen, and the result was that out of one hundred cases there was not one single case affected by any of the various sequelæ that follow that disease. The suppurative process which very frequently is likely to follow, and almost invariably affects the middle ear, was entirely absent, and there is no question about the value and efficacy of that treatment, and it is now becoming an established factor in the treatment of other diseases. The same is largely true of typhoid fever and some modifications of that disease.

*Dr. Alice Steeves,* Chicago (closing discussion).—I have little to say, except that I did not expect so animated a discussion to follow so small a paper; however, I feel very much gratified. This paper has been the outcome of my own observation and experience. I have reason to believe that there has been infection from improperly cleaned instruments in several cases of which I had the care, and I thought it would be well to bring this matter before this section. I feel that sterilization and cleanliness, especially in schools, is not properly demonstrated to the students. We must have ordinary cleanliness before we can have surgical cleanliness.

